# Impairment of Executive Functions in Premenstrual Syndrome: State or Trait?

**DOI:** 10.1016/j.bpsgos.2026.100730

**Published:** 2026-04-06

**Authors:** Anna Madeleine Gnaiger, Patricia Werlein, Patricia Gruschwitz, Magdalena Siebers, Alina Panzik, Erika Comasco, Esmeralda Hidalgo-Lopez, Belinda Pletzer

**Affiliations:** a Department of Psychology, University of Salzburg, Salzburg, Austria; b Centre for Cognitive Neuroscience, University of Salzburg, Salzburg, Austria; c Department of Women’s and Children’s Health, SciLifeLab, Uppsala University, Uppsala, Sweden; d Department of Psychology, University of Michigan, Ann Arbor, Michigan

**Keywords:** Executive functioning, Hormone sensitivity, Inhibitory control, Premenstrual dysphoric disorder, Premenstrual syndrome, Working memory

## Abstract

**Background:**

The aim of this study was to explore whether cognitive impairments in women with premenstrual syndrome (PMS) and premenstrual dysphoric disorder (PMDD) are phase-dependent or persist across the menstrual cycle. Furthermore, we investigated whether such deficits are limited to working memory (WM) or also extend to inhibitory control (IC).

**Methods:**

A total of 105 regularly cycling women between the ages of 19 and 35 years were tested across 3 menstrual cycle phases (mid-follicular, mid-luteal, and late luteal). Premenstrual symptoms were tracked across 2 cycles. Fifty-one women were allocated to the PMS/PMDD group, and 54 women served as control participants. WM and IC were assessed using the n-back and stop signal task, respectively. Hormone levels were determined using saliva samples.

**Results:**

WM deficits in the PMS/PMDD group emerged under high cognitive load and did not vary by cycle phase. The PMS/PMDD group also had significantly longer stop signal reaction times across all cycle phases, with stop signal reaction times positively correlating with premenstrual symptom severity. There was no significant correlation between performance measures and gonadal hormone levels.

**Conclusions:**

Cognitive impairments in PMS/PMDD appear to be trait-like and affect both WM and IC. Additionally, IC was significantly associated with premenstrual symptom severity. Executive deficits may underlie difficulties in emotion regulation and are not solely attributable to gonadal hormone levels.

## Plain Language Summary

In the days leading up to their period, women with premenstrual syndrome (PMS) and premenstrual dysphoric disorder (PMDD) often experience cognitive symptoms such as memory impairments and difficulties concentrating. In this study, we tested 51 PMS/PMDD women and 54 healthy control participants at three time points during their menstrual cycle (mid-follicular, mid-luteal, late luteal) to evaluate their cognitive performance. Behavioral results revealed that the PMS/PMDD group exhibits poorer working memory and inhibitory control performance regardless of cycle phase, suggesting trait-like deficits. Moreover, salivary gonadal hormone concentration did not differ between groups, and estradiol and progesterone levels did not significantly affect task performance.

Premenstrual dysphoric disorder (PMDD) is the most severe form of premenstrual syndrome (PMS) and is classified as a depressive disorder in the DSM-5 (1). The condition is marked by a plethora of cognitive-affective, physical, and behavioral symptoms that typically emerge in the final week before menses, before gradually subsiding within 3 days after period onset (2,3). Core symptoms include anxiety, affective lability, irritability or anger, and depression. Additionally, individuals may experience changes in sleep patterns and appetite, joint and muscle pain, breast tenderness, abdominal bloating, and fatigue (3,4). Many women with PMS have also reported cognitive difficulties, such as impairments in memory or lack of concentration and motor coordination (5,6).

PMS and PMDD often co-occur with various psychiatric conditions, most commonly mood disorders (7), such as major depressive disorder (MDD) (8, 9, 10) and bipolar disorder (BD) (8,11, 12, 13), as well as anxiety and eating disorders, including generalized anxiety disorder, panic disorder, bulimia, and binge eating disorder (8,14, 15, 16). Many of these disorders are associated with deficits in executive functioning. Executive functions (EFs) are top-down cognitive processes that enable goal-directed behavior, reasoning, and problem-solving, while maintaining focus and self-control (17,18). The 3 fundamental components of EFs are cognitive flexibility, working memory (WM), and inhibitory control (IC) (17). WM is defined as the ability to hold and manipulate information over a short period of time, while IC is a neural mechanism primarily tasked with deliberately suppressing or overriding dominant, automatic, or prepotent responses (19,20). In MDD, behavioral deficits have been identified for inhibition, mental flexibility, attentional processes, and multiple memory domains, including WM and long-term memory (18,21,22). Individuals with anxiety disorders often display attentional biases toward threat and diminished cognitive performance under conditions of high emotional interference (23, 24, 25), as well as episodic memory dysfunction, executive inflexibility, and decreased WM capacity and performance efficiency (18,26, 27, 28, 29). Likewise, BD has been linked to impairments in set-shifting, IC, WM, planning, attention, and verbal fluency (30, 31, 32, 33). In both MDD and BD, executive task performance remained compromised even during euthymic states, suggesting trait-like deficits (32,34, 35, 36, 37).

Thus, the question arises whether EF deficits are also present in PMS/PMDD and whether such impairments are phase-specific or extend throughout the menstrual cycle. WM, particularly the n-back task, has been shown to be impaired in both women with PMS (6,38,39) and women with PMDD (40,41). In a study by Aoki *et al.* (42), the PMDD group exhibited significantly lower response accuracy in the 2-back task during the follicular and late luteal phases compared with the control group. Similarly, Slyepchenko *et al.* (6) found WM impairments in participants with moderate to severe PMS independent of cycle phase, further stating that performance deficits were not only evident in conditions with high cognitive load but could also be seen in 0-back results. Additionally, the study group showed significantly more errors of omission, possibly indicating underlying difficulties in selective attention. Pletzer *et al.* (43) provided further evidence for a null effect of cycle phase on EFs in healthy women, concluding that neither verbal nor spatial performance varied significantly across the menstrual cycle. However, other studies yielded conflicting results, indicating WM impairments may be cycle-phase–dependent or nonexistent at all (38,41,44,45). Findings related to IC are even more inconclusive (46,47). Bannbers *et al.* (48) investigated response inhibition in 14 individuals with PMDD and 13 control individuals, employing functional magnetic resonance imaging (fMRI) to capture brain activity. They found that while neither the number of correct and incorrect responses to go and no-go trials, nor respective reaction times (RTs) differed between study groups or cycle phases, women with PMDD had significantly lower activity in task-related parietal areas during the mid-follicular and late luteal phase. Conversely, data by Yen *et al.* (49) demonstrates that women with PMDD had lower IC during no-go trials in a stop signal task. They also had a lower hit rate and more omission errors during go trials, though these differences were only present in the premenstrual phase and not during the follicular phase.

Because of this body of evidence, we hypothesized impaired EF in women with PMS irrespective of menstrual cycle phase. Furthermore, we proposed that EF impairment increases with the extent of premenstrual symptoms. We recruited women with PMS/PMDD and healthy control participants, assessing premenstrual symptom severity and executive functioning during 3 cycle phases (mid-follicular, mid-luteal, late luteal), employing the n-back task to measure WM capacity (50), and the stop signal task to determine response inhibition (51). Premenstrual symptom severity was assessed using the Daily Record of Severity of Problems (DRSP) (52) and used as an indirect measure of individual hormonal sensitivity, drawing on evidence linking PMS symptoms to differential sensitivity to cyclical hormone fluctuations (43).

## Methods and Materials

### Participants

Participants were nulliparous females between the ages of 18 and 35 with regular menstrual cycles (21–35 days, ≤7 days’ variability) (53). Exclusion criteria included any neurological and endocrine disorders, as well as hospitalization for psychiatric conditions and use of hormonal contraceptives within the last 6 months. All participants provided written consent and received either €100 or course credit as compensation. The study was approved by the Ethics Committee of the University of Salzburg and was compliant with the Declaration of Helsinki.

### Procedure

Participants underwent a repeated-measures design, with the following 3 test sessions being scheduled in 3 different cycle phases and in counterbalanced order: mid-follicular (cycle days ∼6–9), mid-luteal (∼6–9 days after ovulation), and late luteal (∼3 days before menses). Cycle phases were determined via ovulation tests and participants’ past 3 cycle dates, then retrospectively confirmed by salivary hormone analyses (54). Adjustments to session timing were made throughout the study to compensate for discrepancies between expected and actual cycle length. Overall, 104 mid-follicular (mean ± SD cycle day = 7.38 ± 2.15), 102 mid-luteal (mean cycle day = −6.71 ± 1.85), and 89 late luteal sessions (mean cycle day = −1.82 ± 0.86) contributed to the final analyses. Each session included saliva sampling, cognitive tasks (stop signal task, n-back, and emotion regulation [ER] task), and questionnaires. To avoid carry-over effects, each task had 3 different task versions, which were also presented in a counterbalanced manner. Premenstrual symptoms were initially assessed using the premenstrual symptom screening tool and later confirmed through DRSP scores, administered daily for 2 successive cycles (52,55,56). This approach was chosen to aid recruitment and ensure an equal distribution of participants across groups. For the final group allocation, the percent increase in DRSP scores from the mid-follicular to the premenstrual phase was calculated (52). For PMDD, ≥5 DSM-5 symptoms, including ≥1 core symptom, must have increased by >50% in both cycles (57). PMS required ≥3 symptoms increasing ≥50% and ≥1 core symptom increasing ≥30%. More information on the participants, the group allocation, and the study procedure is available in Supplemental Methods.

### Stop Signal Task

The stop signal task comprised one training block and 3 experimental blocks (100 trials each, ∼13 minutes total) (51). Green arrows (left/right) were presented on a white background (go signal). Using their dominant hand, participants pressed the corresponding keyboard keys. In 26% of trials, arrows turned red shortly after appearing, telling participants to withhold their response (stop-signal). The stop-signal delay (SSD) started at 250 ms and was adjusted in steps of 50 ms depending on performance to achieve 50% inhibition success (58). Each block had 74 go trials and 26 stop trials. The main outcome, the stop signal reaction time (SSRT), reflects the time needed to inhibit a planned or ongoing response following a stop-signal. It is estimated using the independent race model and can be computed by subtracting the mean SSD from the nth percentile of go response latencies, with n corresponding to the proportion of failed stop trials (51,59,60).

### n-Back Task

The verbal n-back (50) featured 4 load levels (0-, 1-, 2-, and 3-back) and 3 trial types (targets, lures, and non-lures). Participants were presented with a sequential series of black uppercase letters on a white background and had to either press the left arrow key for yes or the right arrow key for no. In the 0-back level, participants responded yes when the presented letter was an X, otherwise pressing no. In the 1-, 2-, and 3-back levels, participants had to indicate whether the current letter matched the letter presented 1, 2, or 3 trials before, respectively. Response latency and accuracy (proportion of correct responses to targets, lures, and non-lures) were recorded (61). Participants performing at or below chance (accuracy ≤0.50) on any trial type (target, lures, and non-lures) were excluded to eliminate potential confounds from guessing. Further task details are described in the Supplemental Methods.

### Hormonal Assessment

Five saliva samples (2 mL each) were collected via the passive drool method and stored at −20 °C until analysis. To remove any solid particles, samples were centrifuged twice, once for 15 minutes and again for 10 minutes, at 3000 rpm, using an Eppendorf 5702 centrifuge. The resulting supernatant was then pooled across the 5 samples per session to account for pulsatile hormone secretion. Salivary concentrations of estradiol and progesterone were determined using Salimetrics ELISA kits. Each pooled sample was analyzed in duplicate. If the coefficient of variance in a sample exceeded 25%, all samples from that participant were reanalyzed.

### Statistical Analyses

Statistical analyses were performed in R (version 4.2.3) and RStudio (version 2025.05.0). Dependent variables were checked for outliers. Strong outliers, deviating more than 3 SDs from the mean of the respective cycle phase, were excluded. Normality assumptions were evaluated through visual inspection, while homogeneity of variance was checked using Levene’s test. Subsequently, Welch’s two-sample *t* tests and Mann-Whitney *U* tests were employed to assess demographic comparability between the 2 samples. Log transformation was applied to correct right-skewed distributions. State measures were evaluated through linear mixed-effects (LME) models with the lme function of the nlme package (62). All models incorporated the cycle phase, group, and their interaction as fixed effects. The participant number (PNr) was treated as a random effect to control for repeated measurements. Dependent and continuous independent variables were standardized using the scale function, allowing *b* values to be interpreted as standardized effect sizes equivalent to Cohen’s *d*. The specific models are detailed in the Results section. The statistical significance of fixed effects was assessed using the analysis of variance function from the stats package. When applicable, post hoc pairwise comparisons were performed using Tukey tests via the glht function (63) or the emmeans function from the multcomp package (64). When significant interaction terms were detected, separate LME models were fitted for each study group to further examine the role of menstrual cycle phase. Effect sizes are reported as partial eta squared (η_p_^2^). The significance level was set to 95%, and significant results are marked with ∗*p* < .05, ∗∗*p* < .01, and ∗∗∗*p* ≤ .001.

## Results

### Demographics

Participants were between the ages of 19 and 35 (mean, years = 24.35 ± 3.67), with an mean cycle length of 28.70 days, SD ± 2.53. Fifty-four test participants were assigned to the control group and 51 to the PMS/PMDD group, of whom 29 met the PMDD criteria. Study groups were comparable in terms of age, general cognitive ability, education, employment, relationship status, health habits, mental health, and cycle length (all *p* ≥ .094) (Table 1).

**Table 1:** Demographic Data Separated for Control Group and PMS/PMDD Group

Variable	Control Group, *n* = 54	PMS/PMDD Group, *n* = 51
Age, Years	24.26 ± 3.48	24.45 ± 3.89
General Cognitive Ability	94.29% ± 7.90%	92.97% ± 9.48%
Education, Qualification for University Entrance	52 (96%)	48 (94%)
Employment Status, Employed	21 (39%)	18 (36%)
In Relationship	23 (43%)	24 (47%)
Relationship Duration, Years	3.25 ± 2.44	1.86 ± 2.28
Smoking Status, Smokers	2 (4%)	3 (6%)
Beck Depression Inventory	7.45 ± 5.11	9.51 ± 6.34
Beck Anxiety Inventory	9.22 ± 5.92	8.25 ± 5.21
Cycle Duration, Days	28.67 ± 2.54	28.75 ± 2.54
Cycle Day Mid-Follicular	7.72 ± 1.13, *n* = 53	7.04 ± 2.81, *n* = 51
Cycle Day Mid-Luteal	−6.63 ± 1.91, *n* = 51	−6.78 ± 1.80, *n* = 51
Cycle Day Late Luteal	−1.85 ± 0.82, *n* = 46	−1.79 ± 0.91, *n* = 43

Values are presented as *n* (%) or mean ± SD.

PMDD, premenstrual dysphoric disorder; PMS, premenstrual syndrome.

### Gonadal Hormone Levels

Hormone levels fluctuated between 0.21 and 5.91 pg/mL for 17β-oestradiol (mean ± SD = 1.26 ± 0.67) and between 0.40 and 604.67 pg/mL for progesterone (mean = 101.72 ± 100.18) (Table 2). There were no significant differences observed between groups (*p* ≥ .707) and no interactions (*p* ≥ .655). Likewise, we did not find phase differences for estradiol (*F*_2,174_ = 1.30, *p* = .275, η_p_^2^ = 0.05) (Table 3). Tukey-adjusted post hoc comparisons revealed that progesterone was significantly higher during the mid-luteal phase compared with both the mid-follicular and late luteal phases (mid-luteal vs. mid-follicular: *d* = 1.22, *z* = 9.98, *p* < .001∗∗∗) (mid-luteal vs. late luteal: *d* = 0.77, *z* = 5.95, *p* < .001∗∗∗).

**Table 2:** Hormone Concentrations, DRSP Scores, and Behavioral Measures by Group and Cycle Phase

	Control Group			PMS/PMDD Group		
	Mid-Follicular	Mid-Luteal	Late Luteal	Mid-Follicular	Mid-Luteal	Late Luteal
Estradiol, pg/mL	1.16 ± 0.60	1.29 ± 0.61	1.21 ± 0.66	1.18 ± 0.72	1.33 ± 0.44	1.36 ± 0.96
Progesterone, pg/mL	50.88 ± 52.42	176.32 ± 126.43	72.16 ± 63.22	53.03 ± 60.82	166.15 ± 104.23	102.94 ± 103.71
DRSP						
Total	1.71 ± 0.53	1.59 ± 0.48	1.88 ± 0.56	1.49 ± 0.45	1.95 ± 0.71	2.41 ± 0.71
Psychological symptoms	1.95 ± 0.74	1.73 ± 0.65	2.06 ± 0.71	1.62 ± 0.60	2.20 ± 0.90	2.73 ± 0.90
Physical symptoms	1.28 ± 0.35	1.35 ± 0.42	1.57 ± 0.53	1.25 ± 0.38	1.51 ± 0.52	1.84 ± 0.62
Impact	1.79 ± 0.88	1.47 ± 0.68	1.57 ± 0.68	1.44 ± 0.72	1.77 ± 1.03	2.26 ± 0.95
Go-RT, ms	419.85 ± 58.78	412.43 ± 67.43	436.58 ± 82.41	427.24 ± 72.42	435.70 ± 78.99	415.02 ± 70.42
Stop RT, ms	480.21 ± 243.44	443.44 ± 228.94	462.90 ± 210.57	488.50 ± 214.10	457.53 ± 147.64	454.84 ± 240.95
Go Hit, %	97.69% ± 6.97%	98.42% ± 2.24%	98.91% ± 1.18%	98.22% ± 2.38%	98.57% ± 1.68%	95.64% ± 15.28%
Stop Hit, %	49.42% ± 5.13%	51.92% ± 9.01%	50.69% ± 8.71%	50.46% ± 4.33%	50.36% ± 5.40%	50.59% ± 10.78%
SSD, ms	214.91 ± 58.80	208.62 ± 82.20	221.24 ± 78.35	209.66 ± 70.12	216.98 ± 73.95	193.66 ± 71.85
SSRT, ms	192.49 ± 25.89	195.34 ± 23.98	199.39 ± 29.19	205.63 ± 20.43	205.25 ± 24.23	209.39 ± 29.75
Target Accuracy, %	81.50% ± 13.70%	80.10% ± 14.30%	83.10% ± 13.70%	80.50% ± 15.40%	80.30% ± 14.70%	80.40% ± 14.90%
Lure Accuracy, %	80.20% ± 15.70%	79.60% ± 15.80%	81.30% ± 15.20%	78.90% ± 15.30%	80.70% ± 15.20%	76.70% ± 16.20%
Target RT, ms	557.00 ± 107.00	546.00 ± 107.00	540.00 ± 91.50	570.00 ± 106.00	568.00 ± 98.30	567.00 ± 111.00
Lure RT, ms	621.00 ± 125.00	616.00 ± 133.00	613.00 ± 117.00	633.00 ± 126.00	630.00 ± 128.00	636.00 ± 137.00

Values are presented as mean ± SD.

DRSP, Daily Record of Severity of Problems; PMDD, premenstrual dysphoric disorder; PMS, premenstrual syndrome; RT, reaction time; SSD, stop-signal delay; SSRT, stop signal reaction time.

**Table 3:** Statistical Parameters of Cycle Phase, Group, Load, and Session Comparisons

	Cycle Phase		Group		Load		Session		Group × Cycle Phase		Group × Load	
	η_p_^2^	*F*	η_p_^2^	*F*	η_p_^2^	*F*	η_p_^2^	*F*	η_p_^2^	*F*	η_p_^2^	*F*
Estradiol	0.05	*F*_2,174_ = 1.30	<0.01	*F*_1,101_ = 0.14	–	–	–	–	<0.01	*F*_2,174_ = 0.40	–	–
Progesterone	0.51	*F*_2,175_ = 50.33∗∗∗	<0.01	*F*_1,103_ = 0.09	–	–	–	–	<0.01	*F*_2,175_ = 0.42	–	–
SSRT	0.01	*F*_2,182_ = 1.09	0.07	*F*_1,103_ = 7.24∗∗	–	–	<0.01	*F*_1,182_ = 1.50	<0.01	*F*_2,182_ = 0.53	–	–
Target Accuracy	<0.01	*F*_2,972_ = 2.07	0.01	*F*_1,102_ = 0.00	0.32	*F*_3,972_ = 63.09∗∗∗	<0.01	*F*_1,972_ = 2.09	–	–	<0.01	*F*_3,972_ = 3.02∗
Lure Accuracy	<0.01	*F*_2,731_ = 0.44	0.01	*F*_1,102_ = 0.20	0.42	*F*_3,731_ = 85.04∗∗∗	0.05	*F*_1,731_ = 41.00∗∗∗	–	–	<0.01	*F*_3,731_ = 0.22
Target RT	<0.01	*F*_2,970_ = 0.29	0.02	*F*_1,102_ = 1.73	0.55	*F*_3,970_ = 191.97∗∗∗	0.08	*F*_1,970_ = 81.83∗∗∗	–	–	<0.01	*F*_3,970_ = 0.32
Lure RT	<0.01	*F*_2,731_ = 0.04	<0.01	*F*_1,102_ = 0.03	0.49	*F*_3,731_ = 110.27∗∗∗	0.15	*F*_1,731_ = 133.81	–	–	<0.01	*F*_3,731_ = 0.50

∗*p* < .05, ∗∗*p* < .01, ∗∗∗*p* < .001.

RT, reaction time; SSRT, stop signal reaction time.

### Stop Signal Task and PMS/PMDD

After excluding outliers, the effectiveness of the task tracking algorithm was validated by inspecting behavioral measures (Table 2). The mean hit rate for stop trials was 50.57% ± 7.45%, indicating proper algorithm function. SSRT was negatively correlated with SSD (*rs*_286_ = −0.36, *p* < .001∗∗∗). The correlation with go-RT was not significant (*rs*_284_ = −0.11, *p* = .058).

**Figure 1:**
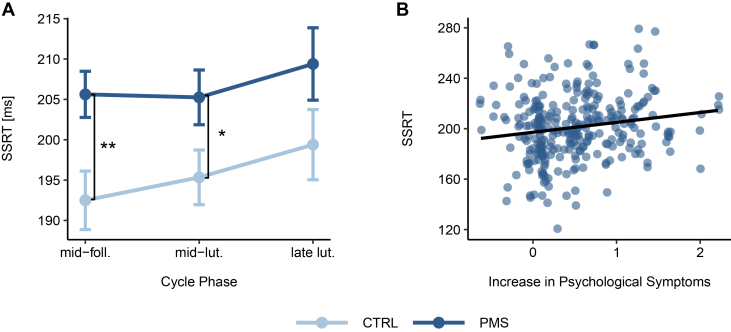
Stop-signal reaction time (SSRT) throughout the menstrual cycle **(A)** and the association between SSRT and psychological symptom increase from the follicular to the luteal phase **(B)**. Depiction of the SSRT **(A)** for the control group (CTRL) and the premenstrual syndrome (PMS)/premenstrual dysphoric disorder (PMDD) group throughout the menstrual cycle including mid-follicular phase (mid-foll.), mid-luteal phase (mid-lut.), and late luteal phase (late lut.). The PMS/PMDD group displays elevated SSRTs in comparison to the control group and independent of menstrual cycle phase **(A)**. Performance differences are the strongest in the mid-foll. phase and smallest in the late lut. phase. SSRT values are positively associated with the absolute increase in psychological symptoms from the follicular to the luteal phase of the menstrual cycle **(B)**. Error bars represent standard errors, and asterisks indicate statistical significance. ∗*p* < .05, ∗∗*p* < .01.

The LME model (SSRT ∼ 1 | PNr + session + phase × group) showed no significant interaction between cycle phase and study group (*F*_1,182_ = 0.53, *p* = .589, η_p_^2^ < 0.01). Hence, the interaction term was removed. In the final model, neither the main effect of testing session nor that of cycle phase was significant (*p* ≥ .223). However, there was a substantial group difference between PMS/PMDD and control participants (*F*_1,103_ = 7.72, *p* = .007∗∗, η_p_^2^ = 0.07) (Figure 1A). An LME model, which replaced group status with PMS severity (i.e., absolute increase in psychological DRSP symptoms from the follicular to the luteal phase), showed similar results (*F*_1,103_ = 4.46, *p* = .037∗, η_p_^2^ = 0.04) (Figure 1B). The model assessing the effect of hormones on SSRT (SSRT ∼ 1 | PNr + session + group + estradiol + progesterone) did not return significant results (estradiol: *F*_1,175_ = 0.09, *p* = .764, η_p_^2^ < 0.01; progesterone: *F*_1,175_ = 0.14, *p* = .710, η_p_^2^ < 0.01).

### n-Back Task and PMS/PMDD

#### Accuracy

**Figure 2:**
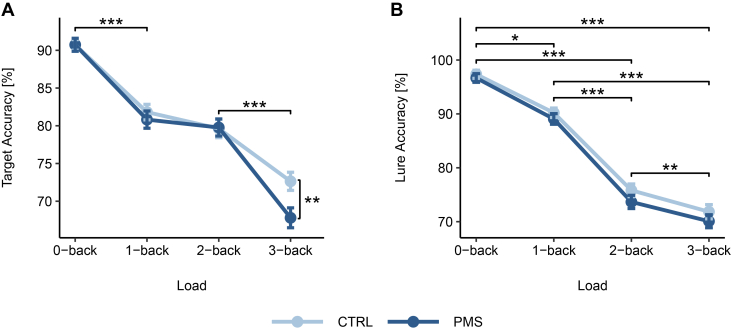
n-Back accuracy in different load conditions and separated by study group. Depicts accuracy for targets **(A)** and lures **(B)** for the control group (CTRL) and premenstrual syndrome (PMS)/premenstrual dysphoric disorder (PMDD) group. n-Back accuracy is reported as a percentage (%), with higher accuracy scores indicating better performance. Target trials show visible group differences in the 3-back condition **(A)**. Additionally, increasing cognitive load was associated with a decline in accuracy for both target **(A)** and lure **(B)** trials. Error bars represent standard errors, and asterisks indicate statistical significance. ∗*p* < .05, ∗∗*p* < .01, and ∗∗∗*p* ≤ .001.

After excluding nonsignificant interaction terms (*p* ≥ .387), the target accuracy model (accuracy ∼ 1| PNr + session + phase + load × group), showed a significant main effect of load (*F*_3,972_ = 63.09, *p* < .001∗∗∗, η_p_^2^ = 0.32) and load group interaction (*F*_3,972_ = 3.02, *p* = .02∗, η_p_^2^ < 0.01). No session (*p* = .149) or phase (*p* = .127) effects emerged. Post hoc pairwise comparisons of adjacent load levels within the control group revealed target accuracy dropped significantly from 0-back to 1-back (*b* = −0.64, SE = 0.10, *t*_500_ = −6.70, *p* < .001∗∗∗) and 2-back to 3-back levels (*b* = −0.54, SE = 0.10, *t*_500_ = −5.34, *p* < .001∗∗∗), but not between 1-back and 2-back conditions (*b* = −0.15, SE = 0.10, *t*_500_ = −1.58, *p* = .305). PMS/PMDD participants initially displayed a sharper decline in accuracy from the 0-back to the 1-back level than control participants (*b* = −0.68, SE = 0.09, *t*_469_ = −7.54, *p* < .001∗∗∗), but showed almost no decrease between 1-back (mean = 80.81 ± 13.31) and 2-back trials (mean = 79.77 ± 13.08; *b* = −0.09, SE = 0.09, *t*_469_ = −0.93, *p* = .727) (Figure 2A). This was followed up by a sizable drop in task performance between the 2-back and 3-back condition (*b* = −0.84, SE = 0.10, *t*_469_ = −8.84, *p* < .001∗∗∗), with PMS/PMDD individuals obtaining significantly lower accuracy scores than the control group (*d* = 0.35, *t*_240_ = 2.7114, *p* = .007∗∗) (Table 3). When swapping study group with PMS severity, only the main effect of n-back load remains (*F*_3,972_ = 154.12, *p* < .001∗∗∗, η_p_^2^ = 0.32; all other *p* ≥ .114), indicating target accuracy was not significantly influenced by PMS severity. Estradiol and progesterone levels did not affect target accuracy (accuracy ∼ 1 | PNr + session + estradiol + progesterone + load × group; estradiol: *F*_1,942_ = 0.21, *p* = .646, η_p_^2^ < 0.01; progesterone) (*F*_1,942_ = 0.12, *p* = .730, η_p_^2^ < 0.01).

To evaluate lure accuracy, we employed the same statistical approach as described above. Main effects for load and session were significant (*F*_3,731_ = 85.04, *p* < .001∗∗∗, η_p_^2^ = 0.42; *F*_1,731_ = 41.00, *p* < .001∗∗∗, η_p_^2^ = 0.05). The interaction between load and study group was not significant (*F*_3,731_ = 0.22, *p* = .881, η_p_^2^ < 0.01), and neither were the main effects for group and cycle phase (*p* ≥ .645). In both groups, lure accuracy progressively decreased as WM load increased, though the decline was slightly more pronounced in the PMS/PMDD group (Figure 2B).

#### Reaction Time

The LME model assessing RT to targets (RT ∼ 1 | PNr + session + phase + load × group) did show significant main effects of load (*F*_3,970_ = 191.97, *p* < .001∗∗∗, η_p_^2^ = 0.55) and session (*F*_1,970_ = 81.83, *p* < .001∗∗∗, η_p_^2^ = 0.08). The interaction of load and study group was not significant (*F*_3,970_ = 0.32, *p* = .811, η_p_^2^ < 0.01). Similar results were obtained for lure trials (Tables 3 and 4). RTs decreased across sessions and became longer as WM load increased, irrespective of group or menstrual phase.

**Table 4:** Values of n-Back Performance Indices by Group and Load Condition

	Control Group				PMS/PMDD Group			
	0-Back	1-Back	2-Back	3-Back	0-Back	1-Back	2-Back	3-Back
Target Accuracy, %	90.72% ± 8.54%	81.81% ± 12.29%	79.63% ± 14.50%	72.64% ± 13.59%	90.73% ± 10.30%	80.81% ± 13.31%	79.77% ± 13.08%	67.80% ± 14.16%
Lure Accuracy, %	97.35% ± 3.12%	90.21% ± 10.72%	75.82% ± 14.19%	71.84% ± 14.95%	96.67% ± 3.43%	89.06% ± 11.72%	73.66% ± 13.75%	70.08% ± 13.54%
Target RT, ms	477.05 ± 66.15	511.49 ± 74.95	595.77 ± 97.98	617.41 ± 96.97	491.73 ± 62.72	532.26 ± 78.06	623.27 ± 98.13	643.07 ± 95.20
Lure RT, ms	445.52 ± 61.65	559.22 ± 105.29	668.83 ± 110.08	648.59 ± 122.17	434.57 ± 61.88	569.74 ± 103.26	695.78 ± 111.58	669.28 ± 120.96

Values are presented as mean ± SD.

PMDD, premenstrual dysphoric disorder; PMS, premenstrual syndrome; RT, reaction time.

## Discussion

In this study, we addressed whether women with PMS/PMDD display executive dysfunction, specifically in WM and IC, and whether these deficits are trait-like or cycle phase-dependent. Additionally, we explored whether impairments in EFs vary as a function of PMS severity. Our findings revealed that the PMS/PMDD group had significantly longer SSRTs in the stop signal task (Table 4). Further analyses demonstrated that SSRTs positively correlated with premenstrual symptom severity. n-back data indicated a greater decline in accuracy under high WM load in individuals with PMS/PMDD compared with control individuals. For both tasks, dysfunctions were not limited to the symptomatic premenstrual phase but persisted throughout the menstrual cycle. Study groups did not differ in their gonadal hormone levels, and estradiol and progesterone did not show significant associations with EFs.

### Gonadal Hormone Levels

Hormone trajectories were as expected, with a peak in progesterone during the mid-luteal phase and no phase differences in estradiol levels, indicating that testing sessions were adequately scheduled or not, e.g., during ovulation. Current research cites individual differences in hormone sensitivity as a central factor in PMS and PMDD etiology (65), proposing that differential responsiveness to normal physiological hormone fluctuations might drive symptom manifestation (66). The fact that we did not detect discrepancies in gonadal hormone levels between study groups agrees with this notion (5,57,66, 67, 68, 69, 70, 71). Our hypothesis that EF impairment would increase with the extent of PMS symptoms was only partially supported; symptom severity was a significant predictor of IC but not WM. Data showed a small positive relationship between PMS/PMDD severity and SSRTs, indicating that individuals with greater symptom exacerbation from the follicular to the luteal phase displayed marginally reduced IC in the stop signal task. No such association was present for the n-back task. Furthermore, analyses did not reveal a significant effect of estradiol and progesterone on IC and WM. This challenges evidence that EFs are influenced by gonadal hormone concentration (72, 73, 74, 75, 76). Nonetheless, our null findings do not necessarily conflict with the broader literature, as hormone fluctuations throughout the menstrual cycle may be too transient and variable to reveal substantial associations (47). The impact of estradiol and progesterone may only become apparent with more pronounced differences, as is the case when comparing menopausal women with and without hormone replacement therapy.

### Inhibitory Control

Results suggest that EF impairment in PMS/PMDD is not limited to WM but extends beyond it to IC. Group differences in IC were the strongest in the mid-follicular phase and least pronounced in the late luteal phase. This aligns with the notion that EF impairment in PMS/PMDD reflects a trait-like vulnerability rather than a state-dependent change. Affected individuals show cognitive deficits across all cycle phases, whereas healthy participants are not impacted or only experience minor declines in IC in the premenstrual phase, specifically narrowing group differences during that time (77). Further evidence stems from a recent MRI study, revealing persistent cortical abnormalities in women with PMDD, alongside smaller changes in cortical structure from the mid-follicular to the late luteal phase in both PMDD and control participants (78).

### Working Memory

For WM, deficits in the patient group were especially apparent when cognitive load was high, indicating reduced WM capacity in PMS/PMDD participants compared with healthy control participants. At low loads, available capacity suffices, but when cognitive demands start exceeding an individual’s updating capability, accuracy begins to decline. In many respects, these results echo findings of earlier studies (6,42,43). Outcomes are further substantiated by neuroimaging data. For instance, previous studies have demonstrated widespread cortical thinning in PMDD patients, especially in the left hemisphere, with many of the affected brain regions implicated in EF, such as the anterior cingulate cortex, inferior and middle frontal gyri, and inferior parietal lobule (79). Beyond cortical thickness, researchers also found reduced whole-brain gray matter volume and gyrification, as well as enhanced connectivity between the left middle temporal gyrus and the left executive control network in women with PMDD, irrespective of cycle phase (78,80,81). Notably, a multimodal investigation using both positron emission tomography and fMRI revealed a substantial increase in dorsal prefrontal cortex (DLPFC) activation in individuals with PMDD during the n-back task in comparison to women without PMDD, with the extent of DLPFC hyperactivation being linked to premenstrual symptoms, disease onset, duration, and burden (40). Since these results occurred under matched hormonal conditions, with all participants undergoing gonadotropin-releasing hormone agonist treatment, followed by either estradiol or progesterone add-back, they point to trait-like neurophysiological differences in how PMDD patients respond to cognitive challenges. Other articles highlighted substantial associations between DRSP scores and cortical thickness, sulcal depth, cortical complexity, and gyrification (82). Moreover, Jeong *et al.* (83) investigated MRI scans of 15 women with PMDD and 15 control participants and found that the patient group showed increased gray matter density in the hippocampal cortex, as well as a decrease in density in the parahippocampal cortex. The authors argued that these abnormalities may indicate functional deficits in limbic and paralimbic regions underlying memory encoding and retrieval, emotional processing, and self-regulation. But since the sample was quite small and gray matter abnormalities were not significantly correlated with premenstrual symptom severity, these findings should be interpreted with caution.

### Relationship With Stress and ER

It is still unclear whether trait impairment of EF is a direct cause of premenstrual symptoms or merely a result of living with the disorder. Persistent deficits in EF may disrupt ER, resulting in amplified stress responses and impaired ability to cope with cycle-related pain. Since PMS and PMDD are still very much underrecognized and diagnosed (84), patients might not recognize their complaints as part of a disorder, leading them to form subjective theories of illness, which could then influence their answers on self-report measures. Research has shown worse ER skills, altered ER strategies, as well as DLPFC dysfunction during emotion regulation task presentation in individuals with PMS/PMDD (81,85, 86, 87, 88, 89). As ER is closely linked to EFs (90), and with deficits in ER appearing state-dependent (91), ER problems may emerge when EF deficits interact with the increased stress and reduced cognitive resources characteristic of the premenstrual phase.

However, impairments in EF could also be caused by heightened responsiveness to stress due to abnormal hormone sensitivity and altered neurophysiology. For instance, Petersen *et al.* (91) reported that the ER ability of PMDD women negatively correlated with perceived stress. Other articles provided similar outcomes (86,92, 93, 94). Moreover, PMS/PMDD women exhibit increased acoustic startle and stress responses (91,95). Since chronic stress exposure and hypothalamic-pituitary-adrenal axis hyperactivation are implicated in structural changes within brain regions such as the prefrontal cortex, hippocampus, and amygdala (96), as well as a decline in cognitive performance (97), the elevated stress levels seen in women with PMS and PMDD might lead to permanent alterations in EF systems.

### Limitations

Primarily recruiting on a university campus resulted in the sample mostly consisting of university students, limiting age, education, and lifestyle variability. The high educational attainment of our sample may explain why significant WM differences emerged only in the most demanding n-back condition, possibly reflecting a ceiling effect and obscuring genuine performance differences. As salivary immunoassays lack the precision of serum-based assessments, hormone measurements were supplemented by backward counting and ovulation kits. Study strengths include the repeated-measures design involving 3 cycle phases, counterbalanced sessions and task versions, prospective PMDD diagnoses, comparability of participants regarding their health habits, lifestyle, and demographic data, the use of ovulation tests and hormone assessments to verify cycle phases, and adequate sample size based on power analyses.

### Conclusions

Individuals with PMS/PMDD display persistent impairments in WM and IC across the menstrual cycle. Executive functioning was not correlated with gonadal hormone levels, and WM performance was not associated with individual hormone sensitivity, as indexed by premenstrual symptom severity, whereas IC was.

## Supplementary Material

Supplemental Methods and Figures S1–S3

Key Resources Table

## References

[1] American Psychiatric Association (2013).

[2] Freeman E.W. (2003). Premenstrual syndrome and premenstrual dysphoric disorder: Definitions and diagnosis. Psychoneuroendocrinology.

[3] Hantsoo L., Epperson C.N. (2015). Premenstrual dysphoric disorder: Epidemiology and treatment. Curr Psychiatry Rep.

[4] Attieh E., Maalouf S., Richa S., Kesrouani A. (2013). Premenstrual syndrome among Lebanese medical students and residents. Int J Gynaecol Obstet.

[5] Reed S.C., Levin F.R., Evans S.M. (2008). Changes in mood, cognitive performance and appetite in the late luteal and follicular phases of the menstrual cycle in women with and without PMDD (premenstrual dysphoric disorder). Horm Behav.

[6] Slyepchenko A., Lokuge S., Nicholls B., Steiner M., Hall G.B.C., Soares C.N., Frey B.N. (2017). Subtle persistent working memory and selective attention deficits in women with premenstrual syndrome. Psychiatry Res.

[7] Bengi D., Strawbridge R., Drorian M., Juruena M.F., Young A., Frey B.N., Yalin N. (2025). A systematic review and meta-analysis on the comorbidity of premenstrual dysphoric disorder or premenstrual syndrome with mood disorders: Prevalence, clinical and neurobiological correlates. Br J Psychiatry.

[8] de Carvalho A.B., Cardoso T.A., Mondin T.C., da Silva R.A., Souza L.D.M., Magalhães P.V.D.S., Jansen K. (2018). Prevalence and factors associated with Premenstrual Dysphoric Disorder: A community sample of young adult women. Psychiatry Res.

[9] Padhy S.K., Sarkar S., Beherre P.B., Rathi R., Panigrahi M., Patil P.S. (2015). Relationship of premenstrual syndrome and premenstrual dysphoric disorder with major depression: Relevance to clinical practice. Indian J Psychol Med.

[10] Wittchen H.U., Becker E., Lieb R., Krause P. (2002). Prevalence, incidence and stability of premenstrual dysphoric disorder in the community. Psychol Med.

[11] Cirillo P.C., Passos R.B.F., Bevilaqua M.C.N., López J.R.R.A., Nardi A.E. (2012). Bipolar disorder and premenstrual syndrome or premenstrual dysphoric disorder comorbidity: A systematic review. Braz J Psychiatry.

[12] Sharma V., Mazmanian D., Eccles H. (2022). Relationship of premenstrual dysphoric disorder with bipolar disorder: A systematic review. J Clin Psychiatry.

[13] Slyepchenko A., Minuzzi L., Frey B.N. (2021). Comorbid premenstrual dysphoric disorder and bipolar disorder: A review. Front Psychiatry.

[14] Breaux C., Hartlage S., Gehlert S. (2000). Relationships of premenstrual dysphoric disorder to major depression and anxiety disorders: A re-examination. J Psychosom Obstet Gynaecol.

[15] Kim D.R., Gyulai L., Freeman E.W., Morrison M.F., Baldassano C., Dubé B. (2004). Premenstrual dysphoric disorder and psychiatric co-morbidity. Arch Womens Ment Health.

[16] Landén M., Eriksson E. (2003). How does premenstrual dysphoric disorder relate to depression and anxiety disorders?. Depress Anxiety.

[17] Diamond A. (2020). Executive functions. Handb Clin Neurol.

[18] Warren S.L., Heller W., Miller G.A. (2020). The structure of executive dysfunction in depression and anxiety. J Affect Disord.

[19] Diamond A. (2012). Executive functions. Annu Rev Psychol.

[20] Funahashi S., Andreau J.M. (2013). Prefrontal cortex and neural mechanisms of executive function. J Physiol Paris.

[21] Murrough J.W., Iacoviello B., Neumeister A., Charney D.S., Iosifescu D.V. (2011). Cognitive dysfunction in depression: Neurocircuitry and new therapeutic strategies. Neurobiol Learn Mem.

[22] Snyder H.R. (2013). Major depressive disorder is associated with broad impairments on neuropsychological measures of executive function: A meta-analysis and review. Psychol Bull.

[23] Armstrong T., Olatunji B.O. (2012). Eye tracking of attention in the affective disorders: A meta-analytic review and synthesis. Clin Psychol Rev.

[24] Boehme S., Ritter V., Tefikow S., Stangier U., Strauss B., Miltner W.H.R., Straube T. (2015). Neural correlates of emotional interference in social anxiety disorder. PLoS One.

[25] Van Bockstaele B., Verschuere B., Tibboel H., De Houwer J., Crombez G., Koster E.H.W. (2013). A review of current evidence for the causal impact of attentional bias on fear and anxiety. Psychol Bull.

[26] Airaksinen E., Larsson M., Forsell Y. (2005). Neuropsychological functions in anxiety disorders in population-based samples: Evidence of episodic memory dysfunction. J Psychiatr Res.

[27] Majeed N.M., Chua Y.J., Kothari M., Kaur M., Quek F.Y.X., Ng M.H.S. (2023). Anxiety disorders and executive functions: A three-level meta-analysis of reaction time and accuracy. Psychiatry Res Commun.

[28] Moran T.P. (2016). Anxiety and working memory capacity: A meta-analysis and narrative review. Psychol Bull.

[29] Sharp P.B., Miller G.A., Heller W. (2015). Transdiagnostic dimensions of anxiety: Neural mechanisms, executive functions, and new directions. Int J Psychophysiol.

[30] Dickinson T., Becerra R., Coombes J. (2017). Executive functioning deficits among adults with bipolar disorder (types I and II): A systematic review and meta-analysis. J Affect Disord.

[31] Dixon T., Kravariti E., Frith C., Murray R.M., McGUIRE P.K. (2004). Effect of symptoms on executive function in bipolar illness. Psychol Med.

[32] Soraggi-Frez C., Santos F.H., Albuquerque P.B., Malloy-Diniz L.F. (2017). Disentangling working memory functioning in mood states of bipolar disorder: A systematic review. Front Psychol.

[33] Sweeney J.A., Kmiec J.A., Kupfer D.J. (2000). Neuropsychologic impairments in bipolar and unipolar mood disorders on the CANTAB neurocognitive battery. Biol Psychiatry.

[34] Bora E., Harrison B.J., Yücel M., Pantelis C. (2012). Cognitive impairment in euthymic major depressive disorder: A meta-analysis. Psychol Med.

[35] Gonda X., Pompili M., Serafini G., Carvalho A.F., Rihmer Z., Döme P. (2015). The role of cognitive dysfunction in the symptoms and remission from depression. Ann Gen Psychiatry.

[36] Olley A.L., Malhi G.S., Bachelor J., Cahill C.M., Mitchell P.B., Berk M. (2005). Executive functioning and theory of mind in euthymic bipolar disorder. Bipolar Disord.

[37] Paelecke-Habermann Y., Pohl J., Leplow B. (2005). Attention and executive functions in remitted major depression patients. J Affect Disord.

[38] Aoki M., Suzuki M., Okayama H. (2020). Assessing n-back task performance of menstrual adult women: Comparison with and without premenstrual syndrome. J Nurs Sci Eng.

[39] Morgan M., Rapkin A.J., D’Elia L., Reading A., Goldman L. (1996). Cognitive functioning in premenstrual syndrome. Obstet Gynecol.

[40] Baller E.B., Wei S.M., Kohn P.D., Rubinow D.R., Alarcón G., Schmidt P.J., Berman K.F. (2013). Abnormalities of dorsolateral prefrontal function in women with premenstrual dysphoric disorder: A multimodal neuroimaging study. Am J Psychiatry.

[41] Yen J.Y., Chang S.J., Long C.Y., Tang T.C., Chen C.C., Yen C.F. (2012). Working memory deficit in premenstrual dysphoric disorder and its associations with difficulty in concentrating and irritability. Compr Psychiatry.

[42] Aoki M., Suzuki M., Suzuki S., Takao H., Okayama H. (2022). Cognitive function evaluation in premenstrual syndrome during the follicular and luteal phases using near-infrared spectroscopy. Compr Psychoneuroendocrinol.

[43] Pletzer B., Bodenbach H., Hoehn M., Hajdari L., Hausinger T., Noachtar I., Beltz A.M. (2024). Reproducible stability of verbal and spatial functions along the menstrual cycle. Neuropsychopharmacology.

[44] Resnick A., Perry W., Parry B., Mostofi N., Udell C. (1998). Neuropsychological performance across the menstrual cycle in women with and without premenstrual dysphoric disorder. Psychiatry Res.

[45] Man M.S., MacMillan I., Scott J., Young A.H. (1999). Mood, neuropsychological function and cognitions in premenstrual dysphoric disorder. Psychol Med.

[46] Henderson A., Gardani M., Dyker G., Matthews L. (2024). Cognition and behaviour across the menstrual cycle in individuals with premenstrual dysphoric disorder—A systematic review. J Affect Disord.

[47] Le J., Thomas N., Gurvich C. (2020). Cognition, the menstrual cycle, and premenstrual disorders: A review. Brain Sci.

[48] Bannbers E., Gingnell M., Engman J., Morell A., Comasco E., Kask K. (2012). The effect of premenstrual dysphoric disorder and menstrual cycle phase on brain activity during response inhibition. J Affect Disord.

[49] Yen J.Y., Tu H.P., Chen C.S., Yen C.F., Long C.Y., Ko C.H. (2014). The effect of serotonin 1A receptor polymorphism on the cognitive function of premenstrual dysphoric disorder. Eur Arch Psychiatry Clin Neurosci.

[50] Owen A.M., McMillan K.M., Laird A.R., Bullmore E. (2005). N-back working memory paradigm: A meta-analysis of normative functional neuroimaging studies. Hum Brain Mapp.

[51] Verbruggen F., Aron A.R., Band G.P., Beste C., Bissett P.G., Brockett A.T. (2019). A consensus guide to capturing the ability to inhibit actions and impulsive behaviors in the stop-signal task. Elif.

[52] Endicott J., Nee J., Harrison W. (2006). Daily Record of Severity of Problems (DRSP): Reliability and validity. Arch Womens Ment Health.

[53] Fehring R.J., Schneider M., Raviele K. (2006). Variability in the phases of the menstrual cycle. J Obstet Gynecol Neonatal Nurs.

[54] Pletzer B., Noachtar I. (2023). Emotion recognition and mood along the menstrual cycle. Horm Behav.

[55] Steiner M., Macdougall M., Brown E. (2003). The premenstrual symptoms screening tool (PSST) for clinicians. Arch Womens Ment Health.

[56] Bentz D., Steiner M., Meinlschmidt G. (2012). SIPS—Screening-Instrument für prämenstruelle Symptome. Die deutsche Version des Premenstrual Symptoms Screening Tool zur Erfassung klinisch relevanter Beschwerden. Nervenarzt.

[57] Comasco E., Kopp Kallner H.K., Bixo M., Hirschberg A.L., Nyback S., De Grauw H. (2020). Ulipristal acetate for treatment of premenstrual dysphoric disorder: A proof-of-concept randomized controlled trial. Am J Psychiatry.

[58] Hall A., Jenkinson N., MacDonald H.J. (2022). Exploring stop signal reaction time over two sessions of the anticipatory response inhibition task. Exp Brain Res.

[59] Logan G.D., Cowan W.B. (1984). On the ability to inhibit thought and action: A theory of an act of control. Psychol Rev.

[60] Verbruggen F., Chambers C.D., Logan G.D. (2013). Fictitious inhibitory differences: How skewness and slowing distort the estimation of stopping latencies. Psychol Sci.

[61] Meule A. (2017). Reporting and Interpreting Working Memory Performance in n-back Tasks. Front Psychol.

[62] Pinheiro J., Bates D., DebRoy S., Sarkar D., R Core Team (2017). https://CRAN.R-project.org/package=nlme.

[63] Hothorn T., Bretz F., Westfall P. (2008). Simultaneous inference in general parametric models. Biom J.

[64] Lenth R.V. (2025). https://cran.r-project.org/web//packages/emmeans/emmeans.pdf.

[65] Rubinow D.R., Schmidt P.J. (2025). Differential sensitivity: Not more or less. Br J Psychiatry.

[66] Hantsoo L., Payne J.L. (2023). Towards understanding the biology of premenstrual dysphoric disorder: From genes to GABA. Neurosci Biobehav Rev.

[67] Bäckström T., Andreen L., Birzniece V., Björn I., Johansson I.M., Nordenstam-Haghjo M. (2003). The role of hormones and hormonal treatments in premenstrual syndrome. CNS Drugs.

[68] Bixo M., Ekberg K., Poromaa I.S., Hirschberg A.L., Jonasson A.F., Andréen L. (2017). Treatment of premenstrual dysphoric disorder with the GABAA receptor modulating steroid antagonist Sepranolone (UC1010)-A randomized controlled trial. Psychoneuroendocrinology.

[69] Nappi R.E., Cucinella L., Bosoni D., Righi A., Battista F., Molinaro P. (2022). Premenstrual syndrome and premenstrual dysphoric disorder as centrally based disorders. Endocrines.

[70] Rubinow D.R., Schmidt P.J. (2006). Gonadal steroid regulation of mood: The lessons of premenstrual syndrome. Front Neuroendocrinol.

[71] Schmidt P.J., Nieman L.K., Danaceau M.A., Adams L.F., Rubinow D.R. (1998). Differential behavioral effects of gonadal steroids in women with and in those without premenstrual syndrome. N Engl J Med.

[72] Luine V.N. (2014). Estradiol and cognitive function: Past, present and future. Horm Behav.

[73] Sherwin B.B. (2003). Estrogen and cognitive functioning in women. Endocr Rev.

[74] Duff S.J., Hampson E. (2000). A beneficial effect of estrogen on working memory in postmenopausal women taking hormone replacement therapy. Horm Behav.

[75] Kampen D.L., Sherwin B.B. (1994). Estrogen use and verbal memory in healthy postmenopausal women. Obstet Gynecol.

[76] Maki P.M., Rich J.B., Rosenbaum R.S. (2002). Implicit memory varies across the menstrual cycle: Estrogen effects in young women. Neuropsychologia.

[77] Souza E.G.V., Ramos M.G., Hara C., Stumpf B.P., Rocha F.L. (2012). Neuropsychological performance and menstrual cycle: A literature review. Trends Psychiatry Psychother.

[78] Dubol M., Stiernman L., Sundström-Poromaa I., Bixo M., Comasco E. (2024). Cortical morphology variations during the menstrual cycle in individuals with and without premenstrual dysphoric disorder. J Affect Disord.

[79] Dubol M., Stiernman L., Wikström J., Lanzenberger R., Neill Epperson C.N., Sundström-Poromaa I. (2022). Differential grey matter structure in women with premenstrual dysphoric disorder: Evidence from brain morphometry and data-driven classification. Transl Psychiatry.

[80] Stiernman L., Dubol M., Sundström-Poromaa I., Bixo M., Comasco E. (2025). Trait- versus state- grey matter volume alterations in premenstrual dysphoric disorder. BMC Psychiatry.

[81] Petersen N., Ghahremani D.G., Rapkin A.J., Berman S.M., Wijker N., Liang L., London E.D. (2019). Resting-state functional connectivity in women with PMDD. Transl Psychiatry.

[82] Dubol M., Wikström J., Lanzenberger R., Epperson C.N., Sundström-Poromaa I., Comasco E. (2022). Grey matter correlates of affective and somatic symptoms of premenstrual dysphoric disorder. Sci Rep.

[83] Jeong H.G., Ham B.J., Yeo H.B., Jung I.K., Joe S.H. (2012). Gray matter abnormalities in patients with premenstrual dysphoric disorder: An optimized voxel-based morphometry. J Affect Disord.

[84] Halbreich U., Borenstein J., Pearlstein T., Kahn L.S. (2003). The prevalence, impairment, impact, and burden of premenstrual dysphoric disorder (PMS/PMDD). Psychoneuroendocrinology.

[85] Ben Elazar A., Canetti L., Azoulay M., Dan R., Goelman G., Segman R. (2023). Quality of life among university students with premenstrual symptoms: The role of emotion regulation. Eur Psychiatry.

[86] Nasiri F., Sharifi S., Mashhadi A., Sharp R. (2022). Premenstrual syndrome: The role of emotion regulation strategies and trait meta-mood. J Rat-Emo Cognitive-. Behav Ther.

[87] Nayman S., Beddig T., Reinhard I., Kuehner C. (2022). Effects of cognitive emotion regulation strategies on mood and cortisol in daily life in women with premenstrual dysphoric disorder. Psychol Med.

[88] Wu M., Liang Y., Wang Q., Zhao Y., Zhou R. (2016). Emotion dysregulation of women with premenstrual syndrome. Sci Rep.

[89] Petersen N., Ghahremani D.G., Rapkin A.J., Berman S.M., Liang L., London E.D. (2018). Brain activation during emotion regulation in women with premenstrual dysphoric disorder. Psychol Med.

[90] Tabibnia G., Monterosso J.R., Baicy K., Aron A.R., Poldrack R.A., Chakrapani S. (2011). Different forms of self-control share a neurocognitive substrate. J Neurosci.

[91] Petersen N., London E.D., Liang L., Ghahremani D.G., Gerards R., Goldman L., Rapkin A.J. (2016). Emotion regulation in women with premenstrual dysphoric disorder. Arch Womens Ment Health.

[92] Comasco E., Sundström-Poromaa I. (2015). Neuroimaging the menstrual cycle and premenstrual dysphoric disorder. Curr Psychiatry Rep.

[93] Eggert L., Witthöft M., Hiller W., Kleinstäuber M. (2016). Emotion regulation in women with premenstrual syndrome (PMS): Explicit and implicit assessments. Cogn Ther Res.

[94] Reuveni I., Dan R., Segman R., Evron R., Laufer S., Goelman G. (2016). Emotional regulation difficulties and premenstrual symptoms among Israeli students. Arch Womens Ment Health.

[95] Liu Q., Wang Y., Van Heck C.H., Qiao W. (2017). Stress reactivity and emotion in premenstrual syndrome. Neuropsychiatr Dis Treat.

[96] McEwen B.S. (2006). Sleep deprivation as a neurobiologic and physiologic stressor: Allostasis and allostatic load. Metabolism.

[97] Moreira P.S., Almeida P.R., Leite-Almeida H., Sousa N., Costa P. (2016). Impact of chronic stress protocols in learning and memory in rodents: Systematic review and meta-analysis. PLoS One.

