# Supplementary material for: Impairment of Executive Functions in Premenstrual Syndrome: State or Trait?
Source: Biol Psychiatry Glob Open Sci. 2026 Apr 6;6(4):100730. doi: 10.1016/j.bpsgos.2026.100730 (PMC13235494; doi:10.1016/j.bpsgos.2026.100730)
Supplement: Supplemental Methods and Figures S1–S3 [file mmc1.pdf]

SUPPLEMENTARY INFORMATION

Persistent Impairment of Executive Functions in Premenstrual Syndrome: State or Trait?

Gnaiger *et al.*

Supplementary Methods

Power Analyses

A priori power analyses conducted in G*Power version 3.1.9.7 (1) determined that a minimum of 86 participants would be needed to detect group differences of moderate effect size (f = 0.25) with 80% power in a design with 3 repeated measurements. To account for an estimated attrition rate of 25%, we aimed to recruit a total of 108 participants, evenly distributed between the PMS/PMDD and control groups (n = 54 per group).

Participants

Participants for the present study were drawn from the same cohort as Lahnsteiner et al. (2025) and Pletzer et al. (2025) (2-3). They were eligible for inclusion if they were female, nulliparous, aged between 18 and 35 years and had a regular menstrual cycle, which was defined as lasting 21 to 35 days with no more than seven days variability between individual cycle lengths (4). Cycle regularity was established based on participants' self-reports of their past three cycle dates and monitored throughout the study. In addition, participants had not used hormonal contraceptives or other hormonal compounds within the last six months and were not taking any medication that could compromise the validity of the collected data. A history of neurological and endocrine disorders, or hospitalization for psychological conditions within the previous six months, also led to exclusion from the study.

In total, 129 participants were recruited through social media and the University of Salzburg. Promotional material openly stated that the study aimed to investigate PMS and PMDD to encourage participation from affected individuals and ensure balanced enrollment across groups. Out of the 129 participants, ten dropped out after the first test session. An additional 14 had to be omitted due to either overlong or anovulatory cycles (n = 8) or inconsistent or missing DRSP records (n = 6), thus resulting in 105 participants being eligible for analyses. Of these, data from 65 participants included test sessions from all three cycle phases. Among the remaining 41 participants, either the late luteal (n = 22, i.e., 21%) or mid-luteal (n = 18, i.e., 17%) sessions had to be excluded (n = 22) or recoded (n = 18) as they fell outside the predefined cycle phase windows or showed inconsistencies between hormonal measurements and the presumed menstrual cycle stage, leading four participants to enter analyses with two

late luteal and 14 with two mid-luteal test sessions. Following exclusions and recoding, 37 participants had their first visit in the mid-follicular phase, 28 in the mid-luteal phase and 35 in the late luteal phase.

Procedure

Participants were assigned to a 3 (group) by 3 (menstrual phase) by 3 (task version) quasi-experimental study design. During an initial screening visit, test subjects were asked to sign a consent form, provide demographic information and fill out the Premenstrual Symptom Screening Tool (PSST) (5,6), along with several trait questionnaires on mood and emotion regulation, including the Beck Anxiety Inventory (7), the revised Beck Depression Inventory (8) and the Difficulties of Emotion Regulation Scale (9). Fluid intelligence was estimated using the first set of the Advanced Progressive Matrices (APM) (10). Afterwards, three test sessions were scheduled in a counterbalanced order, with each session taking place during a different menstrual cycle phase. One session in the mid-follicular phase (approx. cycle days 6-9, low progesterone and estradiol), another in the mid-luteal phase (approx. 6-9 days after ovulation, high progesterone and estradiol levels) and a third one in the late luteal phase (approx. 3 days before the onset of next menses, declining progesterone and estradiol). Cycle phases were determined via commercially available ovulation tests (Pregnafix®) and participants' past three cycle dates. Based on this information, the average cycle length was calculated and used to estimate the expected onset of next menses. Menstrual cycle phases were determined by either subtracting 1-3 days for the late luteal and 4-10 days for the mid-luteal phase, or by adding 5-10 days to receive the time span for the mid-follicular phase. They were then retrospectively confirmed by next period onset and salivary hormone analyses. If period onset occurred significantly earlier or later than expected, the following testing sessions were adjusted accordingly. For instance, if a first session, which was originally scheduled in the late luteal phase, coincidentally fell within the mid-luteal phase due to a delay in menses onset, the dates of subsequent sessions were adapted to capture the remaining two menstrual cycle phases.

Each session lasted about 60-90 minutes and started with the participants being asked to rinse out their mouth with water. The investigators made sure that the subject was not wearing any lip products, had not drunk anything in the last 5 minutes and had not eaten in the last 30 minutes. On top of that, participants were advised not to consume

any soy products a few hours before testing, since the intake of soy could influence hormone balance (11). After the investigator had taken the first saliva sample, the subjects started with the stop signal task (12), which was followed up by the n-back (13) and the Emotion Regulation Task (for a detailed description and corresponding results see Pletzer et al., 2025) (3), with three more saliva samples being taken in-between. Then participants were asked to fill out an online survey and gave one final saliva sample. The online survey comprised the Emotion Regulation Questionnaire (14), the Emoji Positive and Negative Affect Schedule (15), Positive and Negative Affect Schedule (16), the Perceived Stress Scale (17) and the State Trait Anxiety Inventory (18). All questionnaires were administered through LimeSurvey® (Schmitz, 2012; Hamburg, Deutschland). Tasks were displayed using Presentation® (Systems, 2011, Neurobehavioral Systems, http:// www.neurobs.com/).

Assessment of premenstrual symptoms

Premenstrual symptom screening tool (PSST)

The PSST is a 30 item self-assessment measure, which can be used to make tentative PMS or PMDD diagnoses (5-6). In the beginning, women are asked 'Do you experience some or any of the following premenstrual symptoms which start before your period and stop within a few days of bleeding?'. After that, 14 symptoms are listed (i.e., depressed mood). The symptom list is based on DSM-IV criteria and is divided into core symptoms (items 1-4) and additional symptoms (items 5-14). Furthermore, impairment at work, school and in social situations is measured by five supplementary items (items A-E). 29 have four response categories, ranging from 1 (not at all) to 4 (severe). One item is conceived as a 'yes' or 'no' question. Then participants are asked whether they were ever diagnosed with PMS or PMDD. The usual administration time is 3-5 minutes (6). To be assigned to the PMDD group, individuals had to rate at least one of the core symptoms (i.e., items 1-4) as severe and at least five additional symptoms as moderate or severe (i.e., items 1-14). Moreover, the symptoms had to severely impair the subject in at least one of the listed areas (i.e., items A-E). If five symptoms are scored as moderate to severe yet core psychological symptoms and impact ratings are merely moderate, a milder form of PMS was presumed. Participants who did not meet these criteria, were considered part of the control group.

Daily Rating of Severity of Problems (DRSP)

The *Daily Rating of Severity of Problems* (DRSP) (19) is a self-report instrument used to monitor daily changes in premenstrual symptom severity, as well as their impact on daily functioning. Items are rated on a 6-point Likert scale (0 = not at all, … 5 = extreme). The item set for the DRSP is adjustable, with the minimum number of items corresponding to the eleven PMDD symptoms outlined in the DSM-V. For the present study, 16 items were used in total, including eleven items reflecting DSM-V symptoms and an additional five items to allow for a more comprehensive evaluation of physical complaints. To evaluate symptom severity, the percent increase from the mid-follicular phase (cycle days 6-10) to the premenstrual phase, starting five days before period onset, was calculated over two consecutive cycles and for each individual symptom. To be allocated to the PMDD group, at least five out of the eleven DSM-V symptoms must have increased by more than 50% during the premenstrual phase in both menstrual cycles (20). One of those five items had to be a core psychological symptom (depression, anxiety, mood swings, irritability). For the PMS group, at least three symptoms had to show an increase of at least 50%, and at least one core psychological symptom must have increased by 30% or more. Participants who did not meet either set of criteria were assigned to the control group.

Cognitive paradigms

Stop-Signal task

The SST, a two-choice discrimination task that measures IC, was adopted from Verbruggen et al. (2019) (12). There was one training block, as well as three experimental blocks with 100 trials each. The total administration time was around 13 minutes. Between blocks, the SST was paused, enabling participants to take a little break before pressing the spacebar to resume with the next block. Participants were asked to only use their dominant hand to respond, and to answer as quickly and accurately as possible. Three different versions were constructed and administered in a counterbalanced order to avoid carry-over effects. During the task, a series of left- or right-pointing, green-colored arrows were presented on a white background (go-signal, see **Figure S1**). Participants were instructed to respond to each stimulus by pressing the corresponding arrow key on the keyboard (e.g. left arrow = left arrow key) as quickly as possible. Arrows were displayed for up to 600ms, disappearing earlier when a keypress was registered. Trials were interspaced with a black fixation cross of a

randomly determined duration, ranging from 1250 to 1750ms in steps of 125ms. In 26% of trials, the green arrow changed to red shortly after appearing, indicating a stop-trial. In such cases, participants were told to withhold their planned motor response and wait for the next trial. The stop-signal delay (SSD) between the go-signal (i.e., the green arrow) and the stop-signal (i.e., the arrow changing color to red) started at 250ms and was adjusted in steps of 50ms depending on performance, with delays becoming longer when the previous stop-trial was successful and shorter when unsuccessful. The goal was to narrow-in on the SSD at which an individual was able to inhibit their response in 50% of the stop-trials (21). In total, 74 go-trials and 26 stop-trials, equally split between left- and right-pointing arrows, were completed per block, trial sequence randomized. According to Verbruggen et al. (2019), this number of trials is sufficient to obtain reliable estimates of inhibitory functioning (12).

The main outcome of the SST is the stop signal reaction time (SSRT), which reflects the time needed to inhibit a planned or ongoing response following a stop-signal. As this cannot be directly observed, it is estimated using the independent race model. This theory, introduced by Logan & Cowan (1984), posits a race between go and stop processes, where a response only occurs when the go process concludes before the stop process (i.e., $RT < (SSRT + SSD)$) and vice-versa (i.e., $RT > (SSRT + SSD)$) (22-23). Thus, the SSRT can be computed by subtracting the mean SSD from the nth percentile of go response latencies, with n corresponding to the proportion of failed stop-trials (12).

N-back task

The verbal n-back (13) was previously described in Hidalgo-Lopez & Pletzer (2017) (24) is a measure of executive functioning and captures four distinct WM components – inhibition, updating, speed and performance (25). The task featured four load levels (0-, 1-, 2-, and 3-back) and included three different trial types (targets, lures and non-lures). Participants were presented with a sequential series of black uppercase letters on a white background and had to either press the left arrow key for 'yes' or the right arrow key for 'no' (see **Figure S2**). At the beginning of each session, participants received standardized task instructions and were told to respond as quickly and accurately as possible. They then completed training blocks for the 0-, 2-, and 3-back levels. In the 0-back level, which served as a control condition, participants responded 'yes' when the presented letter was an 'X' and otherwise pressed the right arrow key

for 'no'. In the 1-, 2-, and 3-back levels, participants had to indicate whether the current letter matched the letter presented one, two, or three trials before, respectively. The three task versions, each consisting of 16 blocks (4 blocks per condition) with 20 stimuli per block, were presented in a counterbalanced order across test sessions and cycle phases. Each letter was displayed for 1s, followed by a 1s centered fixation cross. Blocks were ordered in a Latin squares sequence and preceded by a brief instruction screen lasting 6s. Trials were pseudo-randomized, with 20% targets, 65% non-lures and 15% lures. Merely the 0-back, which naturally had no lures, consisted of 20% targets and 80% non-lures.

Four types of responses were obtained from this task: The hit rate (correct responses to targets), the false alarm rate (incorrect responses to non-targets), as well as misses (incorrect responses to targets) and correct negatives (correct responses to non-targets). Additionally, response latency and accuracy were recorded. See **Figure S3** for a graphic depiction of reaction time results.

References

(1) Faul, F., Erdfelder, E., Buchner, A., & Lang, A. G. (2009). Statistical power analyses using G*Power 3.1: tests for correlation and regression analyses. *Behavior research methods*, *41*(4), 1149–1160. https://doi.org/10.3758/BRM.41.4.1149

(2) Lahnsteiner, A., Hidalgo-Lopez, E., Noachtar, I., Hausinger, T., Gnaiger, A., Griesbach, K., Scutelnic, D., Risch, A., & Pletzer, B. (2025). Genetic contributions to premenstrual symptoms: revisiting the role of the ESR1 gene. *medRxiv (Cold Spring Harbor Laboratory)*. https://doi.org/10.1101/2025.01.13.25320449

(3) Pletzer, B., Gnaiger, A. M., Kinzelmann, J., Werlein, P., Rauter, L. & Hidalgo-Lopez, E. (2025). Association and Timing of Changes in Emotion Regulation and Mood along the Menstrual Cycle in Women with Premenstrual Syndrome. *medRxiv (Cold Spring Harbor Laboratory)*. https://doi.org/10.1101/2025.03.18.25324201

(4) Fehring, R. J., Schneider, M., & Raviele, K. (2006). Variability in the phases of the menstrual cycle. Journal of Obstetric, *Gynecologic & Neonatal Nursing*, *35*, 376–384. https://doi.org/10.1111/j.1552-6909.2006.00051.x

(5) Steiner, M., Macdougall, M., & Brown, E. (2003). The premenstrual symptoms screening tool (PSST) for clinicians. *Archives of Women's Mental Health*, *16*(6), 203–209. https://doi.org/10.1007/s00737-003-0018-4

(6) Bentz, D., Steiner, M., & Meinlschmidt, G. (2011). SIPS - Screening-Instrument für prämenstruelle Symptome : Die deutsche Version des Premenstrual Symptoms Screening Tool zur Erfassung klinisch relevanter Beschwerden. *Der Nervenarzt*, *83*(1), 33-39.

(7) Margraf, J., & Ehlers, A. (2007). Beck Angst-Inventar—deutsche Bearbeitung. Frankfurt am Main: Harcourt Test Services.

(8) Hautzinger, M., Keller, F., & Kühner, C. (2009). BDI II - Beck-Depressions-Inventar - Manual, (2. Auflage). Pearson Deutschland GmbH.

(9) Gratz, K. L., & Roemer, L. (2004). Multidimensional Assessment of Emotion Regulation and Dysregulation: Development, Factor Structure, and Initial Validation of the Difficulties in Emotion Regulation Scale. *Journal of Psychopathology and Behavioral Assessment*, *26*(1), 41 54. https://doi.org/10.1023/B:JOBA.0000007455.08539.94

(10) Kratzmeier, H., & Horn, R. (1980). *Raven-Matrizen-Test, Advanced Progressive Matrices.* Beltz Test.

(11) Tapiero, H., Nguyen Ba, G., & Tew, K. D. (2002). Estrogens and environmental estrogens. *Biomedicine & Pharmacotherapy*, *56*(1), 36–44. https://doi.org/10.1016/S0753-3322(01)00155-X

(12) Verbruggen, F., Aron, A. R., Band, G. P., Beste, C., Bissett, P. G., Brockett, A. T., Brown, J. W., Chamberlain, S. R., Chambers, C. D., Colonius, H., Colzato, L. S., Corneil, B. D., Coxon, J. P., Dupuis, A., Eagle, D. M., Garavan, H., Greenhouse, I., Heathcote, A., Huster, R. J., . . . Boehler, C. N. (2019). A consensus guide to capturing the ability to inhibit actions and impulsive behaviors in the stop-signal task. *Elife*, *8*. https://doi.org/10.7554/eLife.46323

(13) Owen, A. M., McMillan, K. M., Laird, A. R., and Bullmore, E. (2005). N-back working memory paradigm: a meta-analysis of normative functional neuroimaging. *Human Brain Mapping*, *25*, 46–59. https://doi.org/10.1002/hbm.20131

(14) Abler, B., & Kessler, H. (2011). ERQ. Emotion Regulation Questionnaire [Verfahrensdokumentation aus PSYNDEX Tests-Nr. 9006192 und Fragebogen]. In Leibniz-Zentrum für Psychologische Information und Dokumentation (ZPID) (Hrsg.), Elektronisches Testarchiv. In. Trier: ZPID.

(15) Beltz, A., Foster, K. T., & Loviska, A. (2020). The Emoji Positive and Negative Affect Schedule (E PANAS). https://doi.org/https://doi.org/10.17605/OSF.IO/ZM2B6

(16) Breyer, B., & Bluemke, M. (2016). Deutsche version der positive and negative affect schedule PANAS (GESIS panel).

(17) Schneider, E. E., Schönfelder, S., Domke-Wolf, M., & Wessa, M. (2020). Measuring stress in clinical and nonclinical subjects using a German adaptation of the Perceived Stress Scale. Int J Clin Health Psychol, 20(2), 173-181. https://doi.org/10.1016/j.ijchp.2020.03.004

(18) Laux, L., Glanzmann, P., Schaffner, P., & Spielberger, C. D. (1981). State Trait Anxiety Inventory (STAI). In.

(19) Endicott, J., Nee, J. & Harrison, W. (2006). Daily Record of Severity of Problems (DRSP): reliability and validity. *Archives Of Women S Mental Health*, *9*(1), 41–49. https://doi.org/10.1007/s00737-005-0103-y

(20) Comasco, E., Kallner, H. K., Bixo, M., Hirschberg, A. L., Nyback, S., De Grauw, H., Epperson, C. N. & Sundström-Poromaa, I. (2020). Ulipristal Acetate for Treatment of Premenstrual Dysphoric Disorder: A Proof-of-Concept Randomized Controlled Trial. American Journal Of Psychiatry, 178(3), 256–265. https://doi.org/10.1176/appi.ajp.2020.20030286

(21) Hall, A., Jenkinson, N., & MacDonald, H. J. (2022). Exploring stop signal reaction time over two sessions of the anticipatory response inhibition task. *Experimental Brain Research*, *240*(11), 3061-3072. https://doi.org/10.1007/s00221-022-06480-x

(22) Logan, G. D., & Cowan, W. B. (1984). On the ability to inhibit thought and action: A theory of an act of control. *Psychological Review, 91*(3), 295–327. https://doi.org/10.1037/0033-295X.91.3.295

(23) Verbruggen, F., Chambers, C. D., & Logan, G. D. (2013). Fictitious Inhibitory Differences: How Skewness and Slowing Distort the Estimation of Stopping Latencies. *Psychological Science*, *24*(3), 352-362. https://doi.org/10.1177/0956797612457390

(24) Hidalgo-Lopez, E., & Pletzer, B. (2017). Interactive Effects of Dopamine Baseline Levels and Cycle Phase on Executive Functions: The Role of Progesterone. *Frontiers in neuroscience*, *11*(403), 1-14. https://doi.org/10.3389/fnins.2017.00403

(25) Ren, M., Xu, J., Li, Y., Wang, M., Georgiev, G., Shen, L., Zhao, J., Cao, Z., Zhang, S., Wang, W., Xu, S., Zhou, Z., Chen, S., Chen, X., Shi, X., Tang, X. & Shan, C. (2023). Neural signatures for the n-back task with different loads: An event-related potential study. *Biological Psychology*, *177*, 108485. https://doi.org/10.1016/j.biopsycho.2023.108485

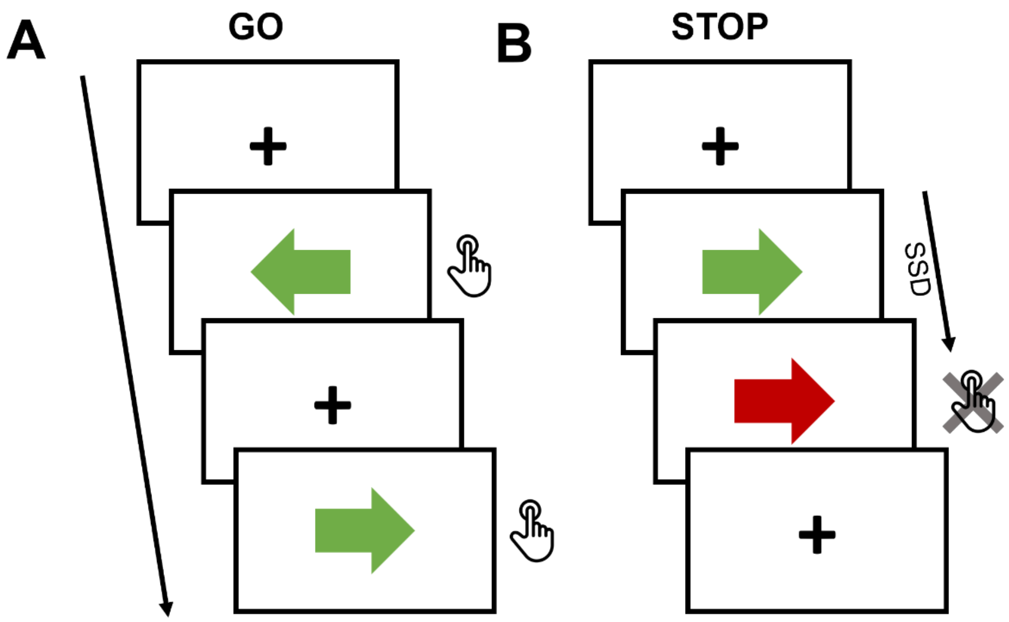

Figure S1. Sequence of events during go- and stop-trials in a stop-signal task. The inter-trial interval was 1250-1750 ms and arrows appeared for up to 600 ms. Stop-signal delay (SSD) was adjusted dynamically using a staircase tracking procedure. In go-trials (A), participants responded by pressing the left or right arrow key according to arrow direction. During stop-trials (B), the arrow changed color from green to red, signaling participants to withhold their response.

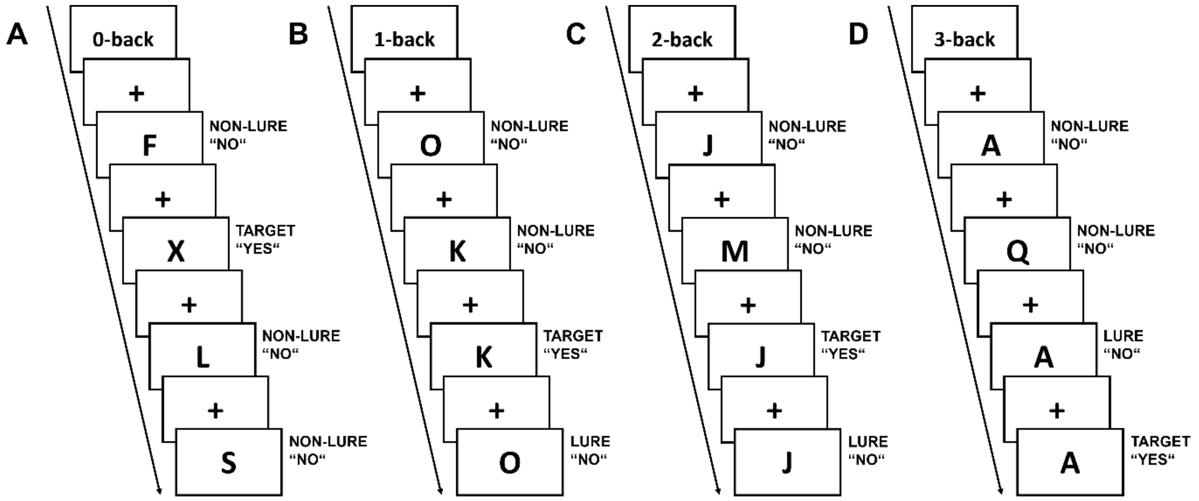

Figure S2. Depiction of four n-back trials. Letters and fixation crosses were displayed for 1 s each. Participants pressed the left arrow key for 'yes' and the right arrow key to respond 'no'. In the 0-back condition (A), the target was the letter X. For the 1-back (B), 2-back (C), and 3-back (D) levels, the current stimulus had to match the letter presented one, two, or three trials before, respectively. Lures closely resembled targets by appearing either one trial too early or one trial too late relative to the correct n-back position. There were no lures in the 0-back condition.

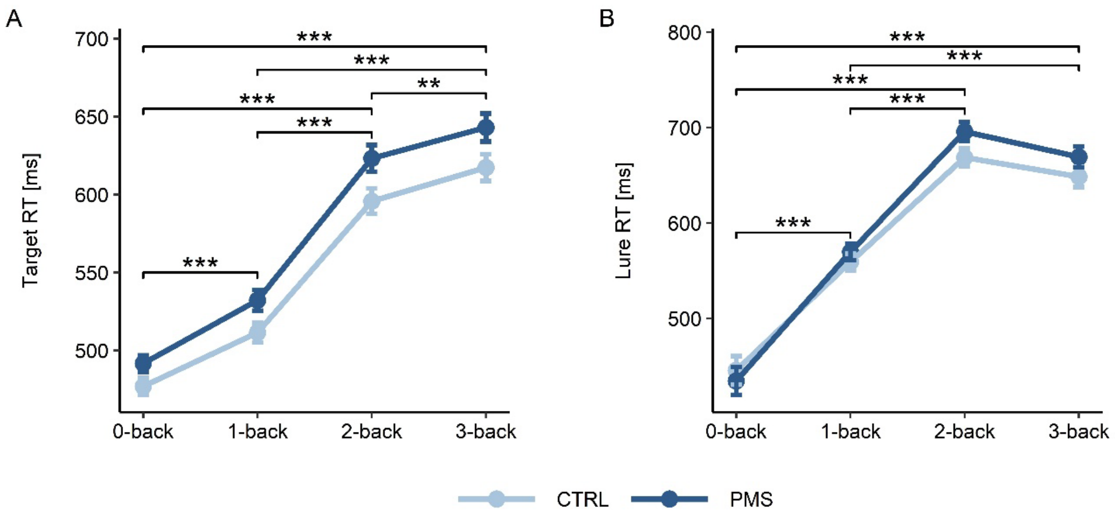

Figure S3. N-back reaction time in different load conditions and separated by study group. N-back reaction times (RT) for targets (A) become slower as cognitive load increases, while lure reaction times (B) also increase before plateauing at higher loads, as suggested by the absence of significant changes between the 2-back and 3-back condition. Moreover, there is no considerable difference in reaction time between the control (CTRL) and PMS/PMDD (PMS) group. Error bars represent standard errors and asterisks indicate statistical significance (* for $p < .05$, ** for $p < .01$, and *** for $p \leq .001$).
